# Gut Microbiome and Common Variable Immunodeficiency: Few Certainties and Many Outstanding Questions

**DOI:** 10.3389/fimmu.2021.712915

**Published:** 2021-08-02

**Authors:** Gilda Varricchi, Remo Poto, Gianluca Ianiro, Alessandra Punziano, Gianni Marone, Antonio Gasbarrini, Giuseppe Spadaro

**Affiliations:** ^1^Department of Translational Medical Sciences, University of Naples Federico II, Naples, Italy; ^2^Center for Basic and Clinical Immunology Research (CISI), University of Naples Federico II, Naples, Italy; ^3^Institute of Experimental Endocrinology and Oncology (IEOS), National Research Council, Naples, Italy; ^4^Department of Internal Medicine and Gastroenterology, Fondazione Policlinico Universitario A. Gemelli IRCCS, Cattolica del Sacro Cuore University, Rome, Italy

**Keywords:** common variable immunodeficiency, fecal microbiota transplantation, inflammation, mucosal immunology, microbiota, probiotics

## Abstract

Common variable immunodeficiency (CVID) is the most common symptomatic primary antibody immunodeficiency, characterized by reduced serum levels of IgG, IgA, and/or IgM. The vast majority of CVID patients have polygenic inheritance. Immune dysfunction in CVID can frequently involve the gastrointestinal tract and lung. Few studies have started to investigate the gut microbiota profile in CVID patients. Overall, the results suggest that in CVID patients there is a reduction of alpha and beta diversity compared to controls. In addition, these patients can exhibit increased plasma levels of lipopolysaccharide (LPS) and markers (sCD14 and sCD25) of systemic immune cell activation. CVID patients with enteropathy exhibit decreased IgA expression in duodenal tissue. Mouse models for CVID unsatisfactorily recapitulate the polygenic causes of human CVID. The molecular pathways by which gut microbiota contribute to systemic inflammation and possibly tumorigenesis in CVID patients remain poorly understood. Several fundamental questions concerning the relationships between gut microbiota and the development of chronic inflammatory conditions, autoimmune disorders or cancer in CVID patients remain unanswered. Moreover, it is unknown whether it is possible to modify the microbiome and the outcome of CVID patients through specific therapeutic interventions.

## Introduction

Elie Metchnikoff, a founder of modern Immunology, suggested that indigenous microbiota provide several pivotal functions for health and disease (1). However, only in recent years, due to the growing access to DNA sequencing technology, important mechanistic insights have been clarified (2). The human gut microbiota consists of 10-100 trillion symbiotic microbes (e.g., bacteria, yeast, and viruses) within each individual (3), whereas the human microbiome encodes over 3 million genes these cells harbor (4). New culture-independent techniques based on high-throughput or next-generation sequencing (NGS) technologies have revolutionized the knowledge of the gut microbiota (5). These approaches have allowed comprehensive analysis of the gut microbiota composition without the limitations of classic culture methods. The most common omics quantification is sequencing of the 16S ribosomal RNA subunit. Such sequencing is relatively simple, but can miss potentially relevant pathobions (6). The whole-community shotgun sequencing (WCS) has been proposed to be a more accurate technique (7). In addition, new omics technologies (e.g., metabolomics and proteomics) go beyond species abundance and have enabled more comprehensive insights on microbiota functions in health and disease (8, 9).

Gut microbiome composition is a dynamic process changing through life. Colonization of the gut begins before delivery (10). The most important contribution to the genesis of the microbiome is vertical transmission of maternal microbiota and the colonization process during delivery (10). Important factors that can affect microbiome composition are type of delivery (vaginal *versus* caesarean) and feeding (formula *versus* breast). Early colonization of the gut microbiome has major effects on its future composition (11). Diet, antibiotics and the environment in early life have an essential role in determining gut microbiota composition in adults (12). For instance, diet can remarkably shape the gut microbiome, making results difficult to interpret in the absence of dietary control.

The gut microbiota plays an important role in educating and modulating the host innate and adaptive immune system (13, 14). The gut microbiome also maintains the intestinal epithelial barrier homeostasis, defense against pathogens (15) and harvests energy from food (16). Germ-free mice, lacking a gut microbial flora, show defects in multiple immune cell populations, such as T_H_2 cells, ILCs and have few IgA-producing intestinal plasma cells, and generally, greater susceptibility to infections (17, 18). Intestinal IgA maturation occurs in response to bacterial colonization of the intestine (19). IgA deficiency causes imbalance of the gut microbiota, resulting in activation of the systemic immune system (20). These findings suggest a close link between gut microbiota, and the local and systemic immune system (21, 22).

Dysbiosis, any change in diversity of gut microbiome, is characterized by loss of beneficial microbes (symbionts) and expansion of potentially pathological organisms (pathobionts) (23). The human gut microbiota is mainly composed of two bacterial phyla: *Bacteroidetes* and *Firmicutes*, which are obligate anaerobic bacteria and constitute approximately 90% of microbial community. Additional phyla include *Actinobacteria*, *Proteobacteria*, *Verrucomicrobia* and *Fusobacteria*. Quantitative and qualitative changes of the gut microbiome are important contributors not only to gastrointestinal disorders but also to systemic metabolic (e.g., obesity, diabetes) and inflammatory diseases (24). There is also evidence for a potential role in health and disease of the viral (*virome*) and fungal community (*mycobiome*) (25).

The aim of this review was to provide an overview of current results and many outstanding questions related to the study of microbiome in common variable immunodeficiency (CVID). We will outline the factors (e.g., decreased secretory IgA, recurrent infections, antibiotics) that shape gut microbiota composition in patients with CVID, and the potential clinical interventions (e.g., diet, probiotics, prebiotics, drugs and fecal microbiota transplantation) to re-establish and/or to promote a “healthier” microbial community.

## Common Variable Immunodeficiency

Common variable immunodeficiency (CVID) is the most common symptomatic primary antibody immunodeficiency (PID) in adulthood with a prevalence of approximately 1/25,000 (26). CVID is characterized by low serum levels of IgG, IgA, and/or IgM and impaired antibody synthesis in response to vaccines and pathogens (27, 28). CVID may present with a wide spectrum of clinical manifestations including increased susceptibility to infections, inflammatory and autoimmune diseases, solid and hematological malignancies (29, 30). The age of onset of CVID varies between 20 and 40 years. The variable age of onset and the heterogeneity of clinical manifestations, which can involve various segments of the respiratory and gastrointestinal tract, is consistent with different phenotypes of this PID.

CVID has complex pathogenic mechanisms resulting from intrinsic or extrinsic defects in B cell differentiation and/or from an impaired cross-talk between B and T cells (31–33). The majority of CVID patients lack a monogenic basis and the disease has probably polygenic inheritance (34). Monogenic disorders involving mutations in genes necessary for B-cell functions represent 2-10% of all CVID patients in different cohorts (35–40).

With the advent of intravenous (i.v.) or subcutaneous (s.c.) immunoglobulin replacement therapy (IgRT), CVID patients are relatively free from life-threatening infections and have a more prolonged survival than several years ago (26). However, IgRT has no proven efficacy in the prevention/treatment of immune dysregulation-related complications (41, 42). It has been estimated that the vast majority (68 to 83%) of CVID patients develop non-infectious complications such as autoimmunity, granulomatous and lymphoproliferative diseases, or malignancies, causing significant morbidity and mortality (29, 42).

It has been suggested that environmental factors (e.g., microbial dysbiosis), *via* epigenetic mechanisms, play a role in inflammatory and immune dysregulation in CVID (43).

Immune dysfunction in CVID can involve different sections of the gastrointestinal tract and cause manifestations including bloating, diarrhea, protein-energy malnutrition and malabsorption (44, 45). Gastrointestinal involvement with malabsorption (=^∼^ 6%), often complicated by nutritional deficiency requiring total parenteral nutrition, is associated with increased mortality and remains a major clinical challenge (42). Moreover, protein-losing enteropathy (PLE) can cause therapeutic failure of the IgRT (46, 47), because circulating IgG, as well as other plasma proteins, are lost through epithelial exudation in the setting of mucosal inflammation and damage (48). Gastrointestinal disorders in CVID can be classified into five groups: infectious (*Giardia lamblia, Cryptosporidium parvum*, *Campylobacter jejuni*, *Salmonella species*, *Clostridioides difficile*, *Cytomegalovirus*, *Norovirus*), small intestinal bacterial overgrowth (SIBO), inflammatory (celiac disease and celiac-like villous atrophy, microscopic colitis, ulcerative colitis, Crohn disease), malignant (gastrointestinal cancers, lymphoma), and autoimmune disorders (pernicious anemia, autoimmune thrombocytopenia and hepatitis, primary biliary cholangitis) (44, 49, 50).

The gastrointestinal tracts involved in CVID display a wide spectrum of histologic patterns. The intestinal mucosa can show increased intraepithelial lymphocytes, villous blunting, crypt distortion, overexpression of apoptosis, paucity of plasma cells and nodular lymphoid hyperplasia, reflecting a mucosal response to gut antigens (51, 52). Among this broad spectrum of gastrointestinal manifestations, CVID-related enteropathy (E-CVID) is characterized by villous atrophy, malabsorption and diarrhea (48, 53).

## GUT Microbiota in CVID

In the last decade, few studies have started to investigate the gut microbiota profile in CVID patients. Gut microbial imbalance of patients with CVID mainly includes changes in microbial diversity, decrease in symbiotic beneficial bacteria, and increase in pathobionts (22, 54–56). The term alpha diversity was introduced to describe the mean microbial species diversity in the gut (43). Higher species diversity leads to higher resilience in the gut ecosystem. In other words, a healthy, resilient gut microbiome relies on high richness and biodiversity (57).

Jørgensen and colleagues performed a 16S ribosomal RNA-based profiling of stool samples in 44 patients with CVID, 45 patients with inflammatory bowel disease (IBD) and 263 healthy controls (22). Alpha diversity of the gut microbiota was reduced in patients with CVID and IBD. CVID patients were also different in beta diversity compared to controls and IBD. Patients with very low serum IgA had reduced alpha diversity. Increased plasma levels of lipopolysaccharide (LPS) were associated with reduced alpha diversity. Two biomarkers [i.e., soluble CD14 (sCD14) and sCD25] of increased microbial translocation and immune cell activation were increased in CVID patients. LPS and sCD25 levels were strong determinants of reduced alpha diversity in a subgroup of patients with inflammatory and autoimmune phenotype. In particular, they found that certain *Firmicutes* of *Clostridia* class (*Lachnospiraceae Dorea* and *Lachnospiraceae Roseburia* families), *Bacilli* and certain *Proteobacteria* of *Gammaproteobacteria* class were increased in CVID. Other *Firmicutes* of *Clostridia* class *(Christensenellaceae* and *Lachnospiraceae Blautia* families)*, Actinobacteria* (*Bifidobacteriaceae* family) and other *Proteobacteria* of *Deltaproteobacteria* class (*Desulfovibrionales* order) were reduced in CVID. Overall, these results, including the decrease of beneficial taxa (e.g., *Bifidobacteriaceae* family*)* and the increase of the detrimental classes *Bacilli* and *Gammaproteobacteria*, emphasize a link between CVID, alterations of gut microbiota, and systemic inflammation.

Shulzhenko et al. examined the duodenal microbiome in 7 patients with E-CVID and 8 patients without enteropathy (54). Both groups had very low levels of serum IgA, but those with enteropathy showed decreased mRNA levels of both IgA subclasses in the duodenal mucosa. IGHA1 mRNA was approximately 10 times higher than IGHA2 and =^∼^ 100 times higher than for IgG genes suggesting that IgA in general and IgA1 in particular is the dominant gut Ig immunoglobulin in CVID small intestine. These data were extended by showing reduced IgA expression, by immunohistochemistry, in the duodenal tissues of CVID patients with enteropathy compared to those without enteropathy. The authors, using transkingdom network analysis of the duodenal microbiome found *Acinetobacter baumannii* as a candidate microbe driving CVID enteropathy. The authors also found that *A. baumannii* activated the monocyte-derived THP-1 cell line, through the expression of interferon (IFN) type I (IFNB1) and CXCL9, with the induction of the Th1-driven inflammation found in CVID enteropathy (54, 58). In addition, type I and type II IFNs were responsible for the shift to pro-inflammatory metabolism in the small intestine. The authors suggested that *A. baumannii* was responsible for villous atrophy and malabsorption in the absence of mucosal IgA. In conclusion, this study suggests that patients with CVID and enteropathy exhibit decreased duodenal IgA expression compared to their counterparts without enteropathy. Moreover, *A. baumannii* exhibited some of the criteria one might expect of an enteropathy-inducing pathobiont.

Using 16S rRNA sequencing, Fiedorová et al. analyzed the bacterial and fungal gut microbiota (mycobiota) in 27 CVID patients and 28 matched healthy controls including 16 case-control pairs living in the same household (55). The alpha diversity of the CVID and control subjects gut community was evaluated in terms of a number of observed OTUs (richness), Shannon index and Chao1 index. All measured alpha diversity indices were lower in the CVID cohort than in controls. In particular, CVID patients with severe phenotype were associated with lower alpha diversity indices. In this study, alterations of alpha diversity were not associated with inflammatory and autoimmune disorders. Interestingly, the authors did not find any significant taxonomic differences of fungal microbiota between patients and controls, suggesting that gut mycobiota may not affect the CVID phenotype. More recently, the same group characterized the fecal microbiota and stool metabolome in a cohort of 6 CVID patients without gastroenterological symptoms and their healthy housemates (56). CVID fecal microbiome showed increased bacterial diversity and differences in several bacterial species compared to controls. Moreover, CVID patients showed in the stool samples decreased levels of adenosine and inosine, two metabolites of purine metabolism, which modulate several aspects of the immune response (59, 60).

The relationships between microbiota-specific systemic IgG and mucosal IgA were examined by Fadlallah et al. (61). These authors demonstrated that serum IgG directed against commensal microbiota are present in healthy subjects and are increased in patients with selective IgA deficiency (SIgAd). Serum IgG and secretory IgA target the same bacteria, and each individual targets a diverse microbiota repertoire. Hence, it is possible to hypothesize that IgRT with IgG preparations from IgA-deficient patients could offer better protection against gut microbial translocations in patients with CVID, especially for treating gastrointestinal-related inflammation (61). In this study, the authors also found that plasma levels of IL-6 and sCD14, two markers of systemic inflammation, were increased in CVID patients compared to healthy controls.

A fundamental question is whether microbiomes in other niches (e.g., oral or respiratory tract), besides gut, can control the immune dysregulation in CVID patients. A recent study highlighted the possible involvement of oral microbiota in CVID (62). The authors showed an increase of oral bacterial load, alpha diversity, and abundance of bacteria from the *Prevotellaceae* family in CVID patients with low serum IgA compared to controls. In addition, decreased serum IgA and expansion of *Prevotellaceae* bacteria were associated with lung disease (62).

The molecular pathways by which gut microbiota contribute to systemic inflammation in patients with CVID are of great importance but remain poorly understood. CVID patients may have increased intestinal permeability caused by a variety of direct and indirect microbial and immunological mechanisms associated to CVID-related enteropathy (44, 48, 63). This condition provides an impairment of the gut barrier, predominantly caused by disruption of intercellular tight junctions. Increased intestinal permeability leads to microbiota-host interactions involving a multitude of bacterial products (e.g., lipopolysaccharides, peptidoglycan, flagellin, RNA, and DNA) released into the portal circulation and influences microbial sensing and systemic immune response through recognition of microbe-associated molecular patterns by the innate immune system (64, 65). On the other hand, gut microbial dysbiosis and infectious triggers may further exacerbate gut leakiness causing increased microbial translocation and inflammation.

Jørgensen and collaborators carefully examined the role of gut microbiota to different aspects of systemic inflammation. In a first study, they confirmed that CVID patients have reduced gut microbial diversity compared to healthy donors (66). Eight-week administration of rifaximin, the oral non-absorbable intestinal antibiotic, did not modify circulating markers (LPS, sCD14, and sCD25) of inflammation in plasma of CVID patients. Rifaximin treatment was associated with a significant change in microbial alpha diversity (Chao1, Shannon index and OTUs). These results support the hypothesis that systemic inflammation in CVID is not influenced by rifaximin-sensitive bacteria. In another study, the same group highlighted the importance of the gut microbiota-dependent metabolite trimethylamine N-oxide (TMAO) in CVID. They found that CVID patients had significantly elevated plasma concentrations of TMAO and trimethylamine (TMA) compared to healthy controls. Increased circulating levels of TMAO correlated with raised LPS and increased inflammatory markers such as TNF-α and IL-12 (67). Plasma TMAO concentrations correlated positively with gut abundance of *Gammaprobacteria.* The authors concluded that TMAO could be a link between gut microbial dysbiosis and systemic inflammation.

In conclusion, there is evidence that the composition of the gut microbiota in patients with CVID is significantly different from healthy individuals (22, 55, 56, 63, 68). Whether changes in the intestinal microbiota observed in CVID are cause or effect of disease still needs to be determined.

Diarrhea is a common gastrointestinal symptom in CVID patients (69–72). Gut-microbial diversity, evaluated by the 16S rRNA gene sequencing, body mass index (BMI), spleen size, and lymphocyte phenotypes were examined in 46 CVID patients reporting ≥ 6 days/months of diarrhea (63). As expected, BMI was lower and malabsorption more common in patients with diarrhea *vs.* non-diarrhea patients. Patients with diarrhea had reduced naïve CD4^+^ T cell counts. Alpha diversity was lower in 15 CVID patients with diarrhea compared to 12 healthy donors. The colonic microbiota of CVID patients with diarrhea differed from that of patients without diarrhea in beta diversity. Alpha diversity was lower in CVID patients compared to controls. There was no difference in alpha diversity between patients with and without diarrhea. Very few CVID patients with diarrhea tested positive for viruses (norovirus, adenovirus, cytomegalovirus, sapovirus). Only one patient was positive for *Clostridioides difficile.*

**Figure 1:**
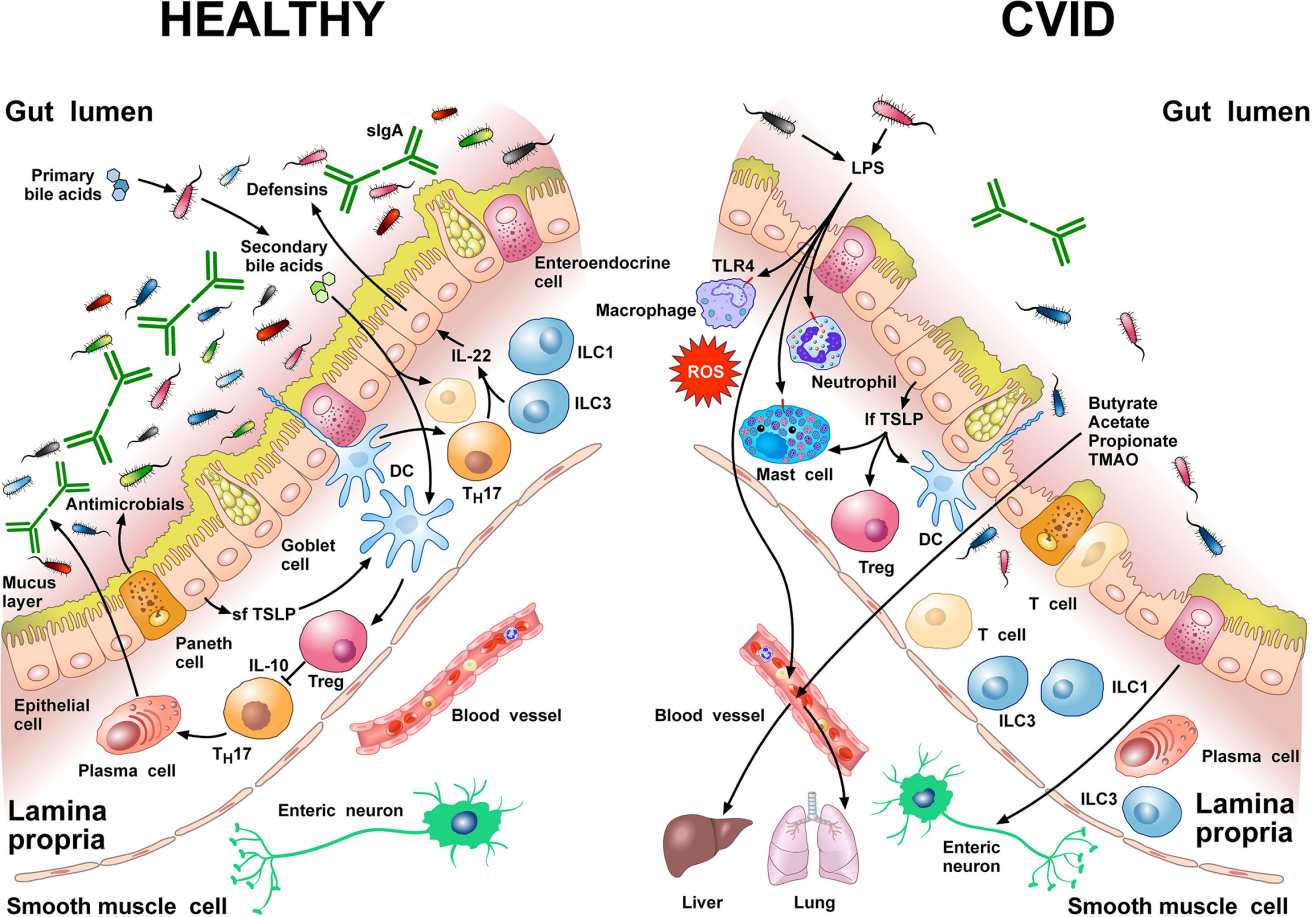
Left side Gut homeostasis and healthy gut are maintained by the interplay between physical barrier (intact mucus layer and epithelial cells) and several cells of the innate and adaptive immune system. In normal subjects four major phyla dominate the gut microbiome: *Bacteriodetes, Firmicutes, Proteobacteria*, and *Actinobacteria.* Dendritic cells (DCs) are pivotal for sensing bacterial products and activating antigen-specific CD4^+^ T cell differentiation. DCs also promote T_H_17 immunity, Foxp3^+^ Treg induction, and IgA production by plasma cells (73). Approximately 5% of primary bile acids transit to the colon and can be metabolized by commensal gut flora (74, 75). These secondary bile acids promote Treg generation and modulate the production of cytokines from DCs (76–78). Several other immune cells (e.g., macrophages, mast cells, neutrophils, ILC3, T_H_1 cells, plasma cells) are sentinels in the mucosal system (79). Neutrophils exert anti-bacterial effects through the release of their stored and newly synthesized mediators and the formation of neutrophils extracellular traps NETs (80). T_H_17 cells produce IL-22 that promotes secretion of anti-microbial peptides such as β-defensins by epithelial cells. ILC1 and ILC3 are present in human intestinal mucosa (81). ILC3 produces IL-22, which plays a role in containing the commensal flora (82) and protecting epithelial cells (83). Paneth cells produce several molecules with antimicrobial activity (α-defensin, REG3, ANG4, sPLA_2_) as well as cytokines that can recruit immune cells (84). The short thymic stromal lymphopoietin isoform (sfTSLP), constitutively expressed by human epithelial cells, is crucial in preserving immune tolerance in the gut (85–87). Right side In patients with CVID, alpha and beta diversity of the gut microbiota is reduced compared to healthy donors (22, 66). Repeated or chronic infections damage the intestinal epithelium (44). The disruption of the gut barrier integrity and the reduction of secretory IgA (54) increase microbial translocation (88) and the permeability of pathogen-associated molecular patterns (PAMPs) such as LPS (22, 66, 89). LPS activates TLR4 on human macrophages (90), neutrophils (91), and mast cells (92, 93) to release pro-inflammatory mediators and ROS. The numbers of ILC3 and ILC1 are abnormally high in the inflamed intestinal mucosa (94). The long TSLP isoform (lfTSLP), induced by several components of gut microbiota, exerts pro-inflammatory effects and contributes to intestinal damage (85–87). The passage of bacteria-derived products (e.g., LPS, butyrate, acetate propionate, TMAO) into the circulation is one of the means of communication between the gut microbiota and the lung or the liver (2, 89, 95, 96). The three most common short chain fatty acids (SCFAs) (butyrate, acetate, and propionate) can also exert immunomodulatory/anti-inflammatory roles (97, 98).

**Figure 1** schematically illustrates some of the structural features and the cells of the innate and adaptive immune system in human colonic mucosa in normal subjects and in CVID patients.

## GUT Microbiota in Experimental CD19 Deficiency

CD19 deficiency is a risk factor for monogenic CVID in humans (99). A mouse model of CVID (CD19^−/−^ mice) is characterized by intestinal malabsorption and defects in lipid metabolism and transport (100). CD19^−/−^ mice develop defective maturation, proliferation, and selection of B cells in the intestinal germinal center, thus resulting in impaired B cell memory and insufficient synthesis of high-affinity antibodies (101) (102, 103). CD19^−/−^ mice had a B cell deficiency in gut-associated lymphoid tissue (GALT) resulting in a dramatic decrease of fecal IgA, IgG, and IgM compared to wild-type (WT) mice. As a consequence, CD19^−/−^ mice cannot bind intestinal bacteria with IgA with subsequent expansion of fecal anaerobic bacteria. These mice develop chronic intestinal malabsorption and altered microbiota composition characterized by outgrowth of anaerobic bacteria within the order *Bacteroidales* (e.g., *Rikenellaceae* and *Lachnospiraceae*), in addition to specific species [*Staphylococcus* spp., *Sutterella* spp., segmented filamentous bacteria (SFB), an undescribed species of alphaproteobacteria, and *Bilophila* spp]. Intestinal malabsorption was associated with intestinal mast cell activation. In this study, treatment with metronidazole and the subsequent reduction in the severity of malabsorption highlights the hypothesis that expansion of anaerobic bacteria was driving the malabsorption in CD19^−/−^ mice. In order to determine whether the intestinal malabsorption was due to dietary gluten exposure, the authors applied a strict gluten-free diet (GFD). Five weeks after initial GFD-exposure the intestinal mucosa histologically showed a reduction of malabsorption. The authors suggested that malabsorption in CD19^−/−^ mice was, at least in part, not only microbiota-dependent but also gluten-sensitive. Of note, gluten sensitivity in patients with CVID is still a matter of debate (100). The results of the previous study suggest that gut microbiota could be an important co-factor in CD19^−/−^ mice. Modification of gluten antigens by microbial transglutaminase may enhance the immunogenicity of gluten peptides, unleashing the inflammatory response (100). In this scenario, the mucosal IgA seems to play an intriguing role as modulators of the gut microbiota (104). In fact, secretory IgA regulates the composition and functions of the gut microbiota by promoting symbiosis between bacteria (105), whereas commensal bacteria induce local IgA response. In addition, IgA binds to members of the commensal gut microbiota and affects colonization levels (106). IgA-coated species include members of the *Proteobacteria* phylum (e.g., *Enterobacteriaceae*) and *Firmicutes* phylum (e.g., *Lactobacilli*), which were relatively increased in the CVID cohort studied by Fiedorová and colleagues. Hence, reduced secretory IgA in CVID patients with enteropathy can lead to microbial dysbiosis.

The use of CD19^-/-^ mice as a clinically relevant model for CVID has been critically examined by Jorgensen and collaborators (107). These authors emphasized that this mouse model does not reflect the polygenic causes of the majority of CVID patients. Therefore, animal models should be carefully considered when studying the interplay between microbiota and immunodeficiency.

## SMALL Intestinal Bacterial Overgrowth and CVID

A recent cross-sectional study on a cohort of 27 CVID individuals reported that symptoms of small intestinal bacterial overgrowth (SIBO) were found in the majority (> 60%) of patients (72). This disorder is characterized by the presence of a pathological concentration of bacteria in the jejunal aspirate equal to or greater than 10^5^ colony-forming units (CFU) per ml. Recognition of this syndrome is made possible by the non-invasive hydrogen tests, such as glucose and lactulose breath tests (108). Symptoms traditionally linked to SIBO include bloating, diarrhea, and abdominal pain/discomfort (72). Steatorrhoea is present in more severe cases. SIBO can contribute to or worsen the nutritional status of CVID patients. In fact, bacterial overgrowth can compromise the absorption of proteins, fats, carbohydrates, vitamins, and other micronutrients. For example, B-12 deficiency is caused by the consumption of cobalamin by anaerobes, malabsorption of the vitamin due to competitive binding with cobalamin from bacterially generated metabolites of cobalamin at the ileal receptor, and in more severe overgrowth, mucosal injury involving the binding site (109). The excess of bacteria, in addition to competing for nutrient intake, can also produce toxic metabolites (e.g., bile acids, hydroxylated fatty acids, and other organic acids) and cause direct damage to the enterocytes of the small intestine, leading to increased intestinal permeability (110). Deconjugation of bile acids by intestinal bacteria may result in malabsorption of fat and fat-soluble vitamins with consequent steatorrhoea and fat-soluble vitamin deficiencies. Bacterial synthesis of folic acid may be responsible for the unusual combination of high folate and B12 deficiency (72).

## The GUT-Lung Axis in CVID

There is compelling evidence of bidirectional gut-lung axis in CVID. Several studies have demonstrated that respiratory infections, mostly prevalent in CVID, are associated with a change in the composition of the gut microbiota (111–117). In particular, many respiratory infections, common in CVID patients, are often accompanied by gastrointestinal symptoms (112, 118). In animal models, bacterial infections or intratracheal instillation of LPS led to alterations in the intestinal microbiota (118, 119). On the other side, repeated and/or chronic gastrointestinal infections can damage the intestinal epithelium resulting in an increased microbial translocation (88). Soluble microbial components (e.g., LPS, peptidoglycans) and metabolites (e.g., butyrate, acetate, propionate, TMAO) transported *via* the circulation are one means of communication between the gut microbiota and the lungs (2, 89, 95, 96). Immune cells can also migrate from the intestine to the respiratory tract *via* the circulation (120). Finally, the gut can modulate immunological and non-immunological responses in the lungs *via* host-derived inflammatory mediators (121). Further studies are needed to ascertain the pathophysiological relevance of the routes of communications within the gut-lung axis in CVID.

## The GUT-Liver Axis in CVID

The microbiota-gut-liver axis might be a key player in the pathogenesis of liver involvement in CVID (44), as the passage of bacteria-derived products into the portal circulation can activate specific receptors on cells of innate immunity (e.g., TLR4 receptors on macrophages and monocytes), leading to liver inflammation (96).

The gut microbiota is now considered a “metabolic organ” that not only facilitates harvesting of nutrients and energy from the ingested food but also produces several metabolites (e.g., bile acids) that signal through their cognate receptors to modulate host metabolism (122) and immune system (123). One class of such metabolites, primary bile acids, produced in the liver, is metabolized to secondary bile acids in the intestine by the gut microbiota (74, 75, 122). Secondary bile acids activate the plasma membrane G protein-coupled bile acid receptor 1 (GPBAR1 or TGR5) and the nuclear Farnesoid-X-Receptor (FXR) (74, 123). GPBAR1 and FXR, highly expressed by intestinal epithelial cells, are also present in immune cells, intestinal muscle cells and neurons (123).

The interaction between the bile and the microbiota is bidirectional. Bile acids can modulate the microbiota through the engagement of FXR (124). GPBAR1 and FXR are expressed by macrophages, DCs, NK cells (125–127), and T helper cells (122). The activation of these receptors inhibits several aspects of the inflammatory pathways (74, 123).

It is well established that Foxp3^+^ Treg cells play a fundamental role in maintaining immune homeostasis in the intestinal lamina propria (79, 128, 129). Bile acid metabolites inhibited the differentiation of T_H_17 cells by binding to the transcription factor retinoid-related orphan receptor-*γ*t (ROR*γ*t) (77). These authors also found that the bile acid metabolite isoallolithocholic acid increased the differentiation of Treg cells through the overexpression of Foxp3. More recently, it has been demonstrated that secondary bile acids increased Foxp3 induction acting on DCs to diminish their immunosuppressive properties (76). Ablating the FXR receptor in DCs enhanced the generation of Treg cells *in vitro* and *in vivo*. Collectively, these studies indicate that secondary bile acids are potent inducers of Tregs, suggesting that microbial metabolism of endogenous steroids contributes to immunological balance in the gut.

These results have translational relevance. Song and coworkers have demonstrated that both dietary and microbial factors modulate bile acid composition and colonic Foxp3^+^ Tregs (130). Importantly, genetic deletion of bile acid metabolic pathways in gut symbionts decreased Treg cell population.

Further studies should investigate possible alterations of bile acid metabolites and host immunological homeostasis in CVID patients.

## Low-Grade Inflammation and Cancer in CVID

Several studies reported an increased risk of malignancies in CVID (131–134). Gastric cancer is the leading cause of death in Italian CVID patients (135). The pathophysiological pathways underlying the relationships between CVID and the increased prevalence of cancers in CVID are of paramount importance but not fully understood. It is well established that chronic low-grade inflammation is a driving force of tumor initiation and progression (136, 137). Moreover, inflammatory stimuli such as LPS, activate immune cells (e.g., macrophages, neutrophils) present in tumor microenvironment (90, 91) and play a role in the switch between dormancy and proliferation of metastatic cells (138, 139). CVID patients exhibit immunological evidence of local and systemic inflammation (22, 61, 66, 67). Future studies should evaluate whether the gut microbiome could be used to identify CVID patients who may develop cancer.

The link between *Helicobacter pylori (H. pylori)* and gastric cancer represents a prototype of interactions between chronic low-grade inflammation caused by a pathobiont and cancer (140). *H. pylori* is a pathogen recognized to play a role in gastric tumorigenesis. *H. pylori* infection in CVID patients appears to facilitate the progression to gastric atrophy and cancer with higher rates compared with the general population, and this could be explained by impaired mucosal immune response to pathogens (141). In particular, low mucosal IgA levels (with bactericidal activity against *H. pylori*) and hypochlorhydria may enhance *H. pylori* colonization and mucosal inflammation, thus promoting gastric carcinogenesis (142). *H. pylori* may not be the only microbe implicated in gastric cancer development. Several studies demonstrate differences in the microbiota of individuals with atrophic gastritis and gastric cancer, suggesting that the alteration of gastric microbiome modulates gastric cancer initiation and progression (143–148). *H. pylori* colonization gradually fades in the final steps of gastric carcinogenesis with *H. pylori* frequently absent/undetectable at the cancer stage (143, 149, 150). Of note, progression to gastric cancer in some patients, can occur after *H. pylori* eradication (135). The latter findings highlight the involvement of additional factors (e.g., other components of the gastric microbiota) in the carcinogenesis process. The latter observation is clinically relevant because follow-up strategies targeting gastric cancer secondary prevention cannot rely exclusively on *H. pylori *identification but also on implementation of upper endoscopies in CVID patients. Further studies appear necessary to identify an altered gastric microbiome in CVID patients at risk of developing gastric cancer.

## GUT Microbiota and Antibiotics

Despite adequate i.v. or s.c. IgRT, recurrent respiratory and gastrointestinal infections are the commonest clinical features in CVID (26, 151). Consequently, a significant percentage of CVID patients are frequently treated with antibiotics to control acute infections or as prophylaxis to reduce infection frequency (152). Antibiotics have also detrimental effects inducing profound alterations of the gut microbiota or dysbiosis and development of bacterial resistance (153). Interestingly, some antibiotics might also have beneficial effects on gut microbiota. For example, rifaximin demonstrated to have eubiotic effect on gut microbiota by increasing the abundance of some bacterial species considered to be beneficial in patients affected by different gastrointestinal and liver diseases (154). Early studies reported that rifaximin reduced plasma endotoxin levels in patients with cirrhosis (155, 156). By contrast, rifaximin had no effect on circulating markers of systemic inflammation (sCD14, sCD25 or LPS), whereas decreased microbial alpha diversity in patients with CVID (66). Moreover, none of the ten major bacteria hitherto indicated to differentiate CVID patients and healthy controls, the *CVID specific dysbiosis index* (22), was significantly modified by rifaximin. The *CVID specific dysbiosis index* correlates with circulating biomarkers of systemic inflammation and gut leakage. Hence, it is possible to hypothesize that the lack of *CVID specific dysbiosis index* modification could explain the absence of an anti-inflammatory effect of rifaximin.

The term “gut resistome” describes the collection of genes or genetic material of gut microbiota that confers antimicrobial resistance (157, 158). Microbial genes conferring resistance to antibiotics can be transferred to gut pathogens in a process named horizontal gene transfer (HGT), thus inducing disease in the host (159). Hence, gut microbiota can be considered as a potential reservoir of antibiotic resistance particularly following recurrent antibiotic therapy (160). Antibiotic-induced resistances can be found even years after the drug administration (161–164). These observations are relevant because prolonged or recurrent antibiotic treatments are frequently administered to CVID patients (152). Antibiotic therapies can also induce impairment in an otherwise healthy gut microbiota, thus contributing to *Clostridioides difficile* infection (CDI) (165, 166). Several antibiotics (i.e., clindamycin, cephalosporins, penicillins, and fluoroquinolones) have been associated with the development of CDI (167). Drug-related factors (e.g., antibiotic class, duration, dose and route of administration of therapy) and host-related factors can profoundly influence the microbial composition. This post-antibiotic dysbiosis can be expressed by loss of diversity, loss of crucial taxa, shifts of metabolic pathways, and reduced colonization resistance against invading pathogens. For these reasons targeted therapies with narrow-spectrum antibiotics and shorter treatment courses are advisable in CVID patients.

## Engineered Modalities of Gut Microbiome

**Figure 2:**
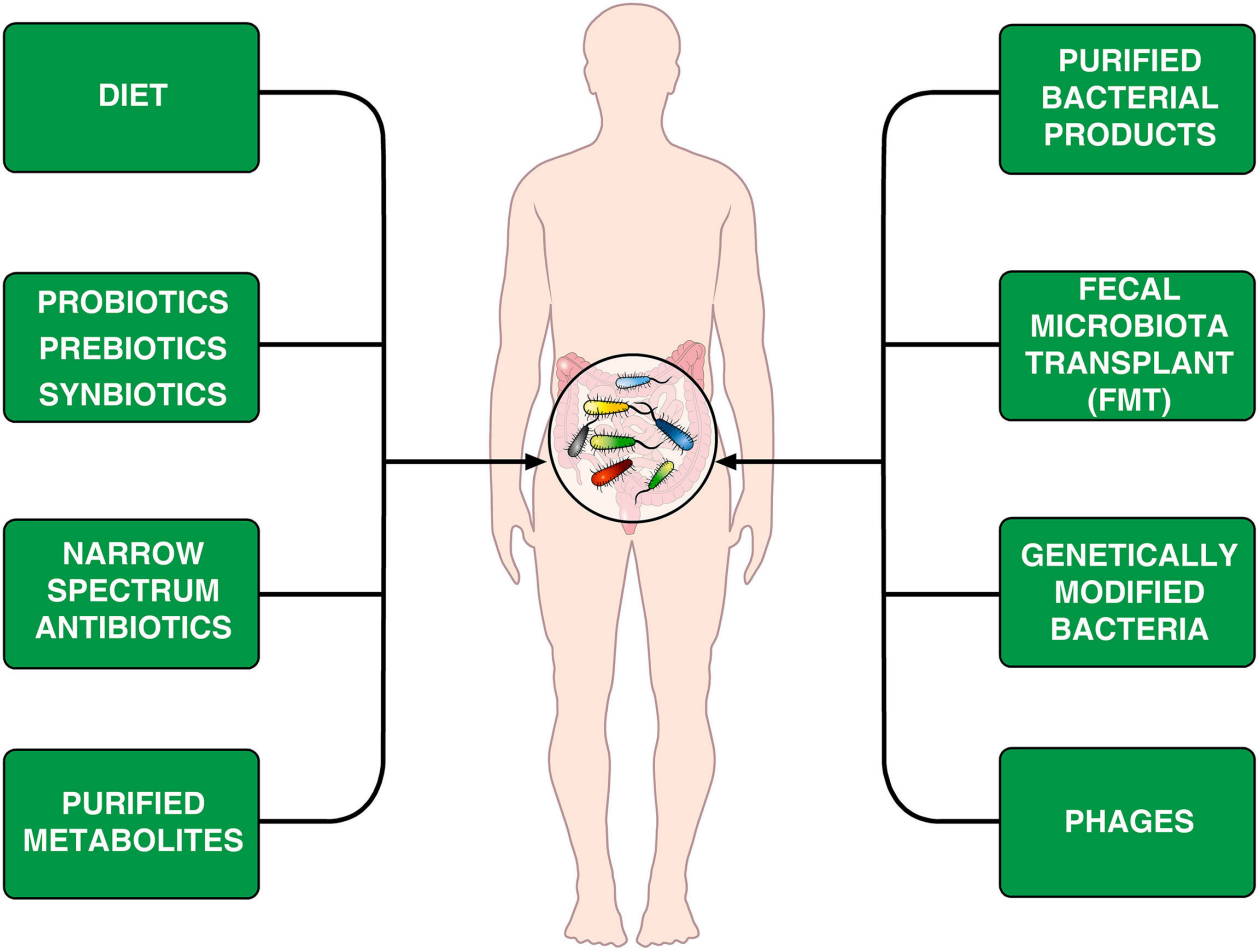
Schematic representation of theoretically therapeutic approaches that can modify the gut microbiome in CVID patients. Diet is an important environmental factor that shapes the microbiota composition (170–172) and can induce Foxp3^+^ Treg cells in the intestine (173). Another approach is the administration of prebiotics, probiotics or synbiotics (174, 175). The different synbiotics, doses, and regimens makes it difficult to perform controlled clinical trials (176). Rifaximin, which is beneficial in patients with certain gastrointestinal diseases (154), decreases alpha diversity in CVID patients without improving markers of systemic inflammation (66). It has been suggested that the administration of specific bacterial products can selectively modulate colonic immune cells in CVID patients (177). Fecal microbiota transplant (FMT), initially developed to treat recurrent *C. difficile* colitis (178, 179) is theoretically an appealing therapeutic tool. Genetically modified bacteria used to treat experimental colitis (180) and phages (181, 182) could theoretically be used for treating microbiome-associated pathologies in CVID patients.

The human microbiological ecosystem changes in response to a variety of environmental stimuli, making difficult to identify specific microbiome signatures in human diseases. Assuming that human microbiome is easily shaped, perhaps we could modulate it to favor a desired outcome (168). The emerging field of microbiome engineering is in its infancy and faces several challenges (169). **Figure 2** provides examples of treatments that can modify the microbiome.

Despite increasing evidence involving gut microbiota in CVID (54, 63, 66, 67), the therapeutic modulation of the gut microbiome has not been investigated yet as a potential treatment for these patients. Elie Metchnikoff first noted that not all microbes are dangerous and envisioned that oral administration of lactic acid-producing bacteria with food (e.g., soured milk) might improve health and longevity (1). Therefore, Metchnikoff was acclaimed as a founding father of contemporary probiotics. There is some evidence that probiotics exert immunomodulatory effects and are considered a promising alternative for the prevention and treatment of certain inflammatory disorders (183). Recent evidence indicates that probiotic-derived metabolites promote differentiation of Foxp3^+^ Tregs (184). The latter observation deserves attention because CVID patients have lower numbers of Treg cell compared to healthy controls in their peripheral blood (185, 186).

## Diet

Dietary contributions to health and chronic conditions, such as obesity, cardiovascular and gastrointestinal disorders, and cancer are of universal importance. Gut microbiome has been implicated as one of several potentially causal human-environment interactions in these disorders (187–189). Host diet plays a fundamental role in shaping the gut microbiota (170–172, 188, 189). Dietary antigens can induce Foxp3^+^ Treg cells in the intestine (173), essential regulators of immunological homeostasis in the colon (190). Diet can modulate the population of colonic Treg cells in mice (130). The relationships between different habitual diets (e.g., healthy plant-based foods, animal-based foods, and butyrate-producer foods), microbiome composition and peripheral and colonic Treg cells in CVID are presently unknown and should be investigated.

## Fecal Microbiota Transplant

Fecal microbiota transplant (FMT), which transfers an entire microbiome from a healthy donor to a recipient, is a therapeutic tool with several potential applications but numerous specific caveats. Transplant can be performed *via* an oral capsule containing fecal extracts (191–193), colonoscopy-guided insertion (193, 194), or enema (195, 196). Healthy donors vary widely in microbiome composition, and donor composition can affect success rates (197, 198). FMT involves risks, and regulatory bodies have recently released safety alerts regarding the risk for transmission of infectious agents *via* FMT (199). Specific guidelines have been released to offer FMT with high levels of safety at the time of COVID-19 pandemic (200).

## Prebiotics, Probiotics, and Synbiotics

Another approach is to administer a formulation of compounds and spores that promote growth of desirable bacteria in the gut. These are referred to as probiotics, although the term refers to formulations containing live microorganisms (201). The term prebiotics refers to compounds (e.g., oligosaccharides and fibers) promoting the growth of specific bacteria (174, 175). The combination of probiotics and prebiotics is called a synbiotic (202). The latter strategy is appealing because one could incorporate any combination of microorganisms to construct a personalized symbiotic (203). The variety of possible synbiotics different formulations, doses, and regimens makes it difficult to make comparisons. The activity of taxonomically similar or even identical strains manufactured by different producers varies. Therefore, the therapeutic properties of a formulation cannot be extrapolated from one preparation to another (203, 204). Moreover, it is not always evident whether gut microbiome can be actually altered by synbiotic regimens. Finally, synbiotics themselves can be associated with adverse events and their safety profile is often overlooked (205, 206). Probiotics, prebiotics, and synbiotics have shown some efficacy in certain experimental (203) and clinical studies (176). However, there is no published evidence addressing these issues in CVID patients.

## Synthetic Microbes

Several bacterial genera have been engineered to produce microorganisms with modified genetic payloads. Steidler et al. developed a genetically modified strain of *Lactococcus lactis* to deliver IL-10 to treat colitis in a mouse model (180). It has been postulated that synthetic bacteria could become in the future a tool for microbiome engineering (207). No successful interventions that utilize engineered microbes to improve patient outcomes have been described.

Phage therapy is another technology for microbiome engineering. The goal is to design phages, infecting and killing undesired bacteria. It has been suggested that phages could serve as a tool for treating microbiome-associated pathologies (181). This approach has been preliminary used since the early twentieth century as a treatment for certain infections (208). Phage therapy has been reported to be successful in a patient infected with multidrug-resistant *A. baumanii* (209). Interestingly, Shulzhenko et al. found *A. baumanii* the microbe driving enteropathy in CVID patients (54).

Although phage therapy is a potential application for microbiome engineering (182), this approach poses several challenges. Phages are live organisms and they could theoretically evolve and cause unknown effects on a microbial community (210).

## Conclusions and Perspectives

In the past decade, several studies have started to identify quantitative and qualitative gut microbiota alterations in CVID patients. It is unclear whether the disruption of eubiotic microbiota observed in CVID is primary or secondary to environmental exposures, antibiotic therapies or diet. Evidence for a role of gut microbiota in the development of local and systemic complications in CVID is still limited. Developments in microbiome field will allow as to shift from a statistical to a casual association research. CVID is not a single disease, but rather a heterogeneous syndrome. Therefore, specific and detailed analyses of the composition (e.g., bacteria, yeast, and viruses) of gut microbiota in different phenotypes of CVID are urgently needed. A common omics quantification of microbiota is sequencing of the 16S ribosomal RNA subunit (22, 54, 63). This is a relatively simple and convenient way to identify organisms in a microbiome, but can miss potentially relevant pathobionts (6, 168). Whole-community shotgun sequencing (WCS) is more accurate to identify individual species and genes (7, 211, 212). We anticipate that advances in sample and sequencing will be critical for further advances in the field. **Table 1** summarizes some of the outstanding pathophysiological questions that should be addressed to better highlight the complex interactions between specific gut microbial species and different phenotypes of CVID.

**Table 1:** Outstanding Pathophysiological Questions.

Is gut dysbiosis observed in CVID patients primary or secondary to disease development? Are specific gut microbial species associated with clinical phenotypes or immunological profiles in CVID? Can gut microbiome be used to identify patients who may develop chronic inflammatory complications, autoimmune disorders or cancer? Are specific genetic or epigenetic defects linked to distinct patterns of microbiome composition in CVID? Are genetic or acquired defects leading to decreased antimicrobial proteins (AMPs) production associated with intestinal inflammation in CVID patients? Are Paneth cell number and antimicrobial functions altered in CVID patients? Although there is evidence linking alterations of bacterial microbiota and CVID, the relationships between viral pathogens and CVID remain scant.

Experimental and clinical studies aimed at modulating gut microbiota in CVID patients are in their infancy. A better knowledge of quantitative and qualitative alterations in pathobionts in these patients is a prerequisite to hypothesize applications from the emerging field of microbiome engineering. A preliminary approach has shown that systemic inflammation in CVID is not modified by the elimination of rifaximin-sensitive bacteria (66). The administration of probiotics, prebiotics or their combination (symbiotics) could represent another strategy to modify the microbiome in CVID patients. FMT, initially developed to treat recurrent *C.difficile* colitis, represents the most radical means of engineering a microbiome (178).

Recently, Geva-Zatorsky and collaborators have devised a sensitive screen that entailed monocolonization of mice with a large number of species of human gut symbionts followed by extensive phenotyping and transcriptomic of immune cells (177). Individual microbes induced colonic T_H_17 cells, Treg cells and DCs or decreased ILCs. These results indicate that future efforts should be devoted to identify bacterial products underlying specific immunomodulatory activity of therapeutic interest. The advantage of the latter approach, using microbial products dosed as a drug, would yield host responses more reproducible overusing whole bacteria to modify gut microbiota.

**Table 2** illustrates some of the outstanding questions concerning the therapeutic corrections of microbiome in CVID patients (213). Further advances in this field might have the potential to revolutionize the approach to prevention and treatment of different phenotypes of CVID.

**Table 2:** Outstanding Therapeutic Questions.

If a microbiome is easily perturbed, could we deliberately alter it to favor a desired outcome of CVID patients? Can gut microbiome engineering lead to reduction of immune dysregulation in CVID patients? Can microbiome engineering in other niches (e.g., oral and upper respiratory tract) lead to control of immune dysregulation in CVID patients? Considering different phenotypes of CVID, which are likely to have advantages from microbiome engineering? Can the type of immunoglobulin replacement therapy (IgRT) (e.g., intravenous *versus* subcutaneous) affect the gut microbiome? Can the gut microbiome affect the short-term and long-term efficacy of subcutaneous or intravenous IgRT in CVID patients?

## Author Contributions

GV, RP, and GS conceived and wrote the manuscript and prepared figures. GI, AP, GM, and AG contributed to the modification and revision of the manuscript. All authors contributed to the article and approved the submitted version.

## Funding

This work was supported in part by grants from the CISI-Lab Project (University of Naples Federico II), TIMING Project and Campania Bioscience (Regione Campania).

## Conflict of Interest

The authors declare that the research was conducted in the absence of any commercial or financial relationships that could be construed as a potential conflict of interest.

## Publisher’s Note

All claims expressed in this article are solely those of the authors and do not necessarily represent those of their affiliated organizations, or those of the publisher, the editors and the reviewers. Any product that may be evaluated in this article, or claim that may be made by its manufacturer, is not guaranteed or endorsed by the publisher.
